# Self-reported diabetes and herpes zoster are associated with a weak humoral response to the seasonal influenza A H1N1 vaccine antigen among the elderly

**DOI:** 10.1186/s12879-019-4214-x

**Published:** 2019-07-23

**Authors:** Manas K. Akmatov, Peggy Riese, Stephanie Trittel, Marcus May, Jana Prokein, Thomas Illig, Christoph Schindler, Carlos A. Guzmán, Frank Pessler

**Affiliations:** 1 Helmholtz Centre for Infection Research, Braunschweig, Germany; 2 Research Group “Biomarkers for Infectious Diseases”, TWINCORE, Centre for Experimental and Clinical Infection Research, Feodor-Lynen-Str. 7, 30625 Hannover, Germany; 3 Centre for Individualised Infection Medicine, Hannover, Germany; 4 Department of Vaccinology and Applied Microbiology, Helmholtz Centre for Infection Research, Braunschweig, Germany; 5 Clinical Research Center Hannover, Hannover Medical School, Hannover, Germany; 6 Hannover Unified Biobank, Hanover Medical School, Hannover, Germany

**Keywords:** Diabetes, Elderly, Fluad®, Herpes zoster, Influenza vaccination

## Abstract

**Background:**

The immune response to seasonal influenza vaccines decreases with advancing age. Therefore, an adjuvanted inactivated trivalent influenza vaccine (Fluad®) exists for elderly individuals. Fluad® is more immunogenic and efficacious than conventional influenza vaccines. However, the immune response varies and may still result in high frequencies of poor responders. Therefore, we aimed to a) examine the prevalence of a weak response to Fluad® and b) identify potential risk factors.

**Methods:**

A prospective population-based study among individuals 65–80 years old was conducted in 2015/2016 in Hannover, Germany (*n* = 200). Hemagglutination-inhibition titers 21 days after vaccination with Fluad® served as indicator of vaccine responsiveness.

**Results:**

The percentage of vaccinees with an inadequate vaccine response varied depending on the influenza strain: it was lowest for H3N2 (13.5%; 95% CI, 9.4–18.9%), intermediate for B strain (37.0%; 30.6–43.9%), and highest for H1N1 (49.0%; 42.2–55.9%). The risk of a weak response to the influenza A H1N1 strain was independently associated with self-reported diabetes (AOR, 4.64; 95% CI, 1.16–18.54), a history of herpes zoster (2.27; 1.01–5.10) and, to a much lesser extent, increasing age (change per year, 1.08; 0.99–1.16). In addition, herpes zoster was the only risk factor for a weak response to the H3N2 antigen (AOR, 3.12; 1.18–8.23). We found no significant association between sex, Body Mass Index, cancer, hypertension, heart attack and CMV seropositivity and a weak response to these two influenza A antigens. Despite its occurence in over one third of vaccinees, none of the variables examined proved to be risk factors for a weak response to the B antigen.

**Conclusions:**

A considerable proportion of elderly individuals displayed a weak vaccine response to this adjuvanted seasonal influenza vaccine and further efforts are thus needed to improve immune responses to influenza vaccination among the elderly. Diabetes and herpes zoster were identified as potentially modifiable risk factors for a poor vaccine response against influenza A antigens, but the results also reveal the need for broader investigations to identify risk factors for inadequate responses to influenza B antigens.

**Trial registration:**

No. NCT02362919 (ClinicalTrials.gov, date of registration: 09.02.2015).

**Electronic supplementary material:**

The online version of this article (10.1186/s12879-019-4214-x) contains supplementary material, which is available to authorized users.

## Background

In the general population, morbidity and mortality from influenza infection is greatest among older individuals, and thus seasonal influenza vaccination is generally recommended to individuals over 60 years of age. However, it is well known that, unfortunately, the risk of mounting a poor immune response to seasonal influenza vaccination also increases with advancing age [1]. This may be associated with age-related alterations in the immune system, a process called immunosenescence [2], as well as memory responses restricted to previously encountered influenza viruses [3, 4]. Apart from immunosenescence, other factors that influence the immune response to conventional (non-adjuvanted) influenza vaccines among the elderly can be divided into the following groups: a) factors related to the individual (e.g. health status, presence of chronic disorders like diabetes and obesity, previous influenza infections or vaccination) [5], b) environmental factors (e.g. chronic cytomegalovirus [CMV] infections, nutritional status, and medication use) [6, 7], and c) molecular factors (e.g. genetic predisposition to a poor immune response) [8, 9].

In order to improve the immune response to influenza vaccines among the elderly, more immunogenic vaccines were recently proposed, including a high-dose vaccine [10, 11], an intradermal vaccine [12], and an adjuvanted vaccine [13]. Fluad® (Novartis Vaccines and Diagnostics S.r.l, Rosia, Italy) is an inactivated trivalent vaccine containing the oil-in-water based adjuvant MF59®, which enhances antigen-dependent immune responses among the elderly [14]. However, this approach still does not induce 100% protection [15]. To date, studies which focus on individual-associated risk factors for an inadequate immune response to Fluad® have been neglected. Thus, the aims of the present study were to a) examine the prevalence of a weak humoral response to Fluad® in an elderly population and b) assess whether there are subgroups of elderly individuals with a higher risk of mounting a weak humoral response.

## Methods

### Study design and study population

A detailed description of the study design including recruitment mechanisms and response rates has been published recently [16, 17]. In brief, we conducted a population-based prospective study in 2015/2016 (*n* = 200). The study participants, males and females between 65 and 80 years of age, were randomly selected from the resident’s registration office in Hannover, Germany. After giving informed consent, participants were immunized intramuscularly with the trivalent adjuvanted subunit vaccine Fluad®. The vaccine contained HA antigens from influenza B virus and influenza A strains H1N1 and H3N2 adapted to the 2015/16 influenza season. Blood samples were collected before (day 0) and 1/3 (one half of the participants on day 1 and from the other half on day 3), 7, 21 and 70 days after vaccination.

### Laboratory analysis

A hemagglutination inhibition (HAI) assay was applied to serum samples collected on day 0 and day 21 or 70 post vaccination. Samples were treated with RDE (receptor destroying enzyme derived from Cholera filtrate, Sigma-Aldrich) at a ratio of 1 volume serum to 4 volumes of RDE and kept at 37 °C overnight. Subsequently, the reaction was stopped by incubation at 56 °C for 30 min. Samples were then mixed with an equal volume of 0.9% NaCl and frozen at − 20 °C until further use. For the HAI assay, serial dilutions of the serum samples were mixed with standardized concentrations of the respective vaccine antigen (A/California/7/09 (H1N1)(NYMC-X181), A/Switzerland/9715293/2013 (H3N2)(NIB88) or B/Brisbane/9/2014 for the 2015/2016 season, NIBSC) and incubated with turkey-derived red blood cells. The HAI titer was defined as the highest serum dilution that still inhibited hemagglutination.

### Determination of CMV serological status

The CMV status of the study participants was assessed using serum samples taken before vaccination (day 0). The concentration of CMV-specific IgG was determined using the “CMV-IgG-ELISA PKS”- kit (medac diagnostika, Wedel, Germany) according to the manufacturer’s protocol. Briefly, diluted serum samples were applied to the pre-coated microtiter plates and CMV-specific IgG antibodies were detected using a peroxidase-coupled anti-human IgG antibody. The subsequent quantification was performed according to the manufacturer’s specifications and quality standards.

### Dependent variables

The humoral response to each of the three antigens (A H1N1, A H3N2 and B) constituted the three dependent variables used in this study. An adequate humoral response to the respective antigen was defined as a ≥ 4-fold HAI titer increase between day 0 and 21, or as a titer ≤10 on day 0 and ≥ 40 on day 21/70 post vaccination. Accordingly, a weak vaccine response was defined as an HAI titer increase < 4-fold or < 40 for individuals with a titer ≤10 before vaccination.

### Independent variables

Basic sociodemographic data (sex, age, and education), data on common infections in the last 12 months (upper and lower respiratory tract infections, gastrointestinal tract infections, labial herpes, infections of the skin and mucosa, and bladder and kidney infections), life-time infections (sepsis, endocarditis, and herpes zoster) and acute and chronic non-communicable diseases (e.g. heart attack, diabetes mellitus, cancer, and asthma) were collected using a self-administered paper-based questionnaire. The question regarding diabetes could not distinguish between type 1 and type 2, but from their epidemiology in the elderly it is expected that most cases correspond to type 2. Information on current medication use was collected using a medication list (the latter was provided by the participants’ primary care physician). Before blood pressure (BP) measurements the study participants had a 5-min rest period. BP was measured three times with pauses of at least half a minute, using the Carescape V100 (GE Healthcare, Berlin, Germany), and the mean of the three measurements was recorded. Hypertension was defined as a systolic or diastolic blood pressure higher than 140 mm Hg and/or 90 mm Hg, respectively [18]. Height and body weight were measured with the devices Seca 222 (SECA, Hamburg, Germany) and MPE 250K100HM (Kern & Sohn, Balingen-Frommern, Germany), respectively. Body Mass Index (BMI) was calculated using the formula “weight (kg)/height^2^ (m)”.

### Statistical analysis

First, we calculated crude prevalence rates of adequate and weak humoral responses to the seasonal influenza vaccine Fluad® and their 95% approximate binomial confidence intervals (CI) according to Wilson [19]. Second, we calculated post-stratification weights with respect to sex and age to obtain nationally representative estimates for the above mentioned prevalences. For this, the sex and age distribution of the respective age groups of the general German population in the respective year were taken from the German Federal Statistical Office [20]. Third, the humoral vaccine (non) response pattern to the three influenza vaccine antigens (A H1N1, A H3N2 and B) was visualized with a Venn diagram. Fourth, we calculated crude and adjusted odds ratios for having a weak humoral response to each of the three vaccine antigens. Multivariable fractional polynomial regression was applied to examine the association between each antigen and sex (female vs. male), age, Body Mass Index and CMV (all three fitted as second-degree fractional polynomial [21]), presence of self-reported diabetes mellitus, cancer, heart attack, herpes zoster, and objectively measured arterial hypertension. We applied a “full model” strategy [22], where the selection of variables of interest was based on literature review [23]. The chi-squared test was used to assess significance of differences between categorical variables. Analyses were performed with Stata, version 12 (StataCorp LP, Texas, USA) and the R Foundation for Statistical Computing, version 3.3.2 (www.r-project.org).

## Results

### Description of the study population

**Fig. 1:**
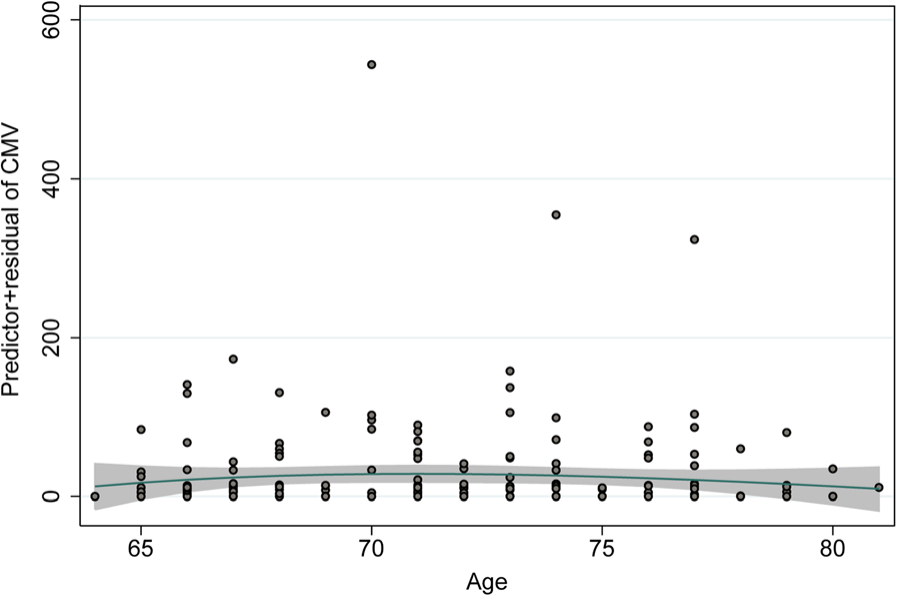
CMV-specific IgG by participants’ age*. * Estimated by fractional polynomials; the best powers for age among 44 models were − 2, −2

The demographic, health-related and laboratory characteristics of the study population stratified by sex are shown in Table 1. Of the 200 participants, more than half of the study participants had hypertension. Forty-five percent and 18% of the participants were overweight and obese, respectively. Fifty-three percent of the participants had a positive CMV status. Figure 1 shows the fractional polynomial function for the relationship between CMV and age; CMV titer increased slightly up to the age of 70 years and decreased afterwards. There were no significant differences across demographic and health-related characteristics between female and male participants; only BMI constituted an exception in that there was a higher proportion of overweight males (55%) than females (32%) (*X*^*2*^ = 13.129, df = 3, *n* = 200, *p* = 0.004).

**Table 1:** Demographic, health-related and laboratory characteristics of the study population, %

Characteristics	Total sample (*N* = 200)	Female (*n* = 85)	Male (*n* = 115)	*p* value (*X*^*2*^ test^d^)
Demographic characteristics				
Age groups				0.949
65–70 y	35	35	35	
71–75 y	37	35	37	
76–80 y	28	29	28	
Health-related characteristics				
Body Mass Index^a^				0.004
Underweight (≤18.49 kg/m^2^)	1.0	2.4	0	
Normal weight (18.50–24.99 kg/m^2^)	36	46	28	
Overweight (25.00–29.99 kg/m^2^)	45	32	55	
Obesity (≥30.00 kg/m^2^)	18	20	17	
Hypertension^b^				0.645
Yes	56	54	57	
No	44	46	43	
Heart attack^c^				0.511
Yes	6.0	4.7	7.0	
No	93	94	92	
Don’t know	0.50	0	0.87	
Missing values	0.50	1.2	0	
Cancer^c^				0.481
Yes	20	18	22	
No	80	81	77	
Don’t know	0	0	0	
Missing values	0	1.2	0.87	
Diabetes mellitus^c^				0.875
Yes	7.5	7.1	7.8	
No	92	91	92	
Don’t know	0.50	1.2	0	
Missing values	0	1.2	0	
Herpes zoster^c^				0.404
Yes	18	20	16	
No	79	77	82	
Don’t know	2.0	1.2	2.6	
Missing values	1.0	2.4	0	
Self-perceived health status^c^				0.071
Poor	0	0	0	
Fair	9.0	12	7.0	
Good	64	64	64	
Very good	21	15	25	
Excellent	4.0	7.1	1.7	
Missing values	2.0	2.4	1.7	
Ever vaccinated against influenza^c^				0.112
Yes	78	80	74	
No	21	17	24	
Don’t know	1.0	2.4	0	
Missing values	1.5	1.2	1.7	
Laboratory parameters (mean ± SD)				
WBC count [x 10^9^L] (normal values)	6.66 ± 1.70	6.91 ± 1.83	6.48 ± 1.59	0.08 ^e^
		(3.5–11)	(3.5–11)	
Neutrophils [x 10^9^L] (normal values)	4.03 ± 1.42	4.18 ± 1.55	3.93 ± 1.30	0.228 ^e^
		(2.0–7.0)	(2.0–7.0)	
Monocytes [x 10^9^L] (normal values)	0.58 ± 0.17	0.57 ± 0.16	0.59 ± 0.17	0.436 ^e^
		(0.2–1.0)	(0.2–1.0)	
Lymphocytes [x 10^9^L] (normal values)	1.89 ± 0.89	1.97 ± 0.69	1.83 ± 1.01	0.247 ^e^
		(1.0–3.0)	(1.0–3.0)	
Erythrocytes [x 10^12^L] (normal values)	4.81 ± 0.40	4.70 ± 0.39	4.89 ± 0.40	0.001 ^e^
		(3.90–5.00)	(4.32–5.72)	
Haemoglobin [g/dL] (normal values)	14.57 ± 1.26	13.95 ± 1.04	15.02 ± 1.22	< 0.0001 ^e^
		(12.0–15.5)	(13.5–17.5)	
Thrombocytes [tsd/μL] (normal values)	225.6 ± 54.1	256.2 ± 53.6	202.8 ± 42.0	< 0.0001 ^e^
		(150–450)	(150–450)	
CMV-specific IgG				0.555
Negative	45	44	46	
0.45–5.00 IU/mL	6.0	3.5	7.8	
5.00–15.00 IU/mL	25	26	24	
> 15.00 IU/mL	24	27	23	

^a^ Weight and height were measured at the study center (see Methods). Body Mass Index (BMI) was calculated using the formula “weight/height^2^” (kg/m^2^)

^b^ Blood pressure was measured at the study center (see Methods). Hypertension was defined as a systolic or diastolic blood pressure ≥ 140 mmHg and/or 90 mmHg, respectively

^c^ Self-reported information

^d^ The category “Don’t know” was not considered for chi-squared test

^e^ t-test for independent data

### Systemic adverse events after vaccination

The most common reported systemic adverse event after vaccination was chills (13%), followed by malaise, headache and sore throat (Table 2).

**Table 2:** Systemic adverse events after an adjuvanted influenza vaccine^a^

Symptoms	n^b^	%
Chills	25	13
Malaise	6	3.0
Headache	5	2.5
Sore throat	4	2.0
Muscle aches	3	1.5
Fatigue	1	0.5

^a^Fluad®, an inactivated trivalent influenza vaccine adjuvanted with MF59® recommended for the respective influenza season

^b^Multiple answers were possible

### Humoral vaccine response patterns

**Fig. 2:**
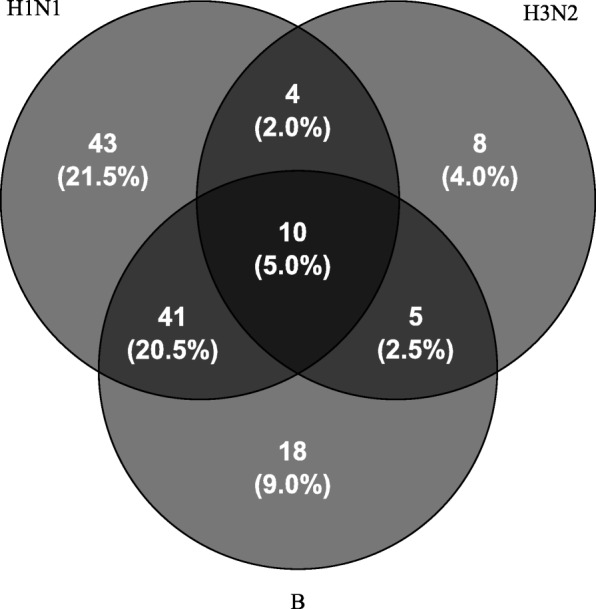
Venn diagram showing common and distinct humoral responses to the three antigens contained in the vaccine (i.e. A H1N1, A H3N2 and B). Percent values do not sum up to 100%; 71 (35.5%) participants had an adequate response to all three antigens

We observed distinct patterns of the vaccine response to the three different vaccine components (Fig. 2). Only 36% of the participants mounted an adequate response to all three vaccine antigens. Thirty-five percent of the study participants did not respond to any one of the antigens, 25% did not respond to any two antigens, and 5% did not respond to all three.

### Prevalence of a weak vaccine response to Fluad®

The crude prevalence rates of weak humoral responses varied depending on influenza strains/subtypes (Table 3). The prevalence rate was lowest for the A H3N2, intermediate for the B, and highest for the A H1N1 component. The weighted prevalence rates differed only marginally from crude prevalence rates (Table 3).

**Table 3:** Crude and weighted prevalence rates of weak humoral response to an adjuvanted influenza vaccine among the elderly^d^

Vaccine strains	Proportion of individuals with a weak humoral response^a^, % (95% CI^b^)	Weighted proportion of individuals with a weak humoral response, %^c^
A H1N1	49.0 (42.2–55.9)	49.3
A H3N2	13.5 (9.4–18.9)	12.5
B	37.0 (30.6–43.9)	36.1

^a^Responders to the respective components of the influenza vaccine are those who had a ≥ 4-fold HAI titer increase between day 0 and day 21 with respect to the time of vaccination. Nonresponders are those who did not have a fourfold titer increase

^b^95% approximate binomial confidence intervals (CI) were calculated according to Wilson

^c^Post-stratification weights were calculated with respect to sex and age to obtain nationally representative estimates. The sex and age distribution of the general German population in the respective year was obtained from the Federal Statistical Office

^d^Fluad®, an inactivated trivalent influenza vaccine adjuvanted with MF59® recommended for the respective influenza season, was used

### Risk factors associated with a weak humoral vaccine response to Fluad®

No differences in the parameters assessed with complete blood count were detected in participants with a weak humoral response as compared to the participants with an adequate humoral response (Additional file 1: Table S1). The results of the multivariable fractional polynomial regression analyses are presented in Table 4. Self-reported diabetes (AOR, 4.64; 95% confidence intervals, 1.16–18.54) and a history of herpes zoster (shingles) (2.27; 1.01–5.10) were the strongest risk factors for a weak humoral response to the influenza A H1N1 strain. In addition, the risk of being an A H1N1 non-responder increased by 8% per year of increased age (1.08; 0.99–1.16), but the results were less significant due to the CI crossing below 1. The study participants with self-reported herpes zoster infection additionally had a 3-fold higher risk of being an A H3N2 non-responder (Table 4, fourth and fifth column). None of the variables were associated with influenza B non-responsiveness in a multivariable model. Of note, CMV positivity was not associated with a poor response to any of the three antigens.

**Table 4:** Risk factors associated with a weak humoral response to each of the three vaccine antigens contained in the adjuvanted influenza vaccine Fluad® among individuals ≥65 years of age^d^

Variables	A H1N1		A H3N2		B	
	UOR & 95% CI	AOR & 95% CI	UOR & 95% CI	AOR & 95% CI	UOR & 95% CI	AOR & 95% CI
Female vs. male	1.21 (0.69–2.13)	1.36 (0.73–2.50)	0.52 (0.22–1.26)	0.48 (0.19–1.20)	0.74 (0.41–1.32)	0.78 (0.42–1.46)
Age (change per year)	1.06 (0.99–1.13)	1.08 (0.99–1.16)	0.99 (0.90–1.09)	1.01 (0.91–1.12)	1.05 (0.98–1.12)	1.03 (0.96–1.11)
BMI (change per unit)^a^	1.02 (0.96–1.08)	1.01 (0.94–1.08)	1.00 (0.91–1.10)	0.99 (0.89–1.09)	0.96 (0.90–1.03)	0.98 (0.91–1.05)
Hypertension^b^	0.73 (0.42–1.28)	0.67 (0.36–1.23)	0.82 (0.37–1.86)	0.94 (0.40–2.19)	1.05 (0.59–1.87)	0.94 (0.51–1.72)
Diabetes^c^	5.11 (1.40–18.53)	4.64 (1.16–18.54)	1.53 (0.41–5.76)	1.16 (0.24–5.65)	0.37 (0.10–1.35)	0.58 (0.14–2.29)
Cancer^c^	1.39 (0.69–2.79)	1.26 (0.58–2.69)	0.88 (0.31–2.50)	0.73 (0.24–2.25)	1.74 (0.86–3.52)	1.57 (0.74–3.31)
Heart attack^c^	3.41 (0.89–13.01)	2.78 (0.64–12.15)	3.54 (0.99–12.71)	3.39 (0.80–14.40)	0.32 (0.07–1.49)	0.44 (0.09–2.21)
Herpes zoster^c^	1.99 (0.94–4.23)	2.27 (1.01–5.10)	2.71 (1.10–6.69)	3.12 (1.18–8.23)	1.03 (0.48–2.20)	0.96 (0.43–2.12)
CMV (change per unit)	1.00 (0.99–1.00)	1.00 (0.99–1.00)	1.00 (0.99–1.01)	1.00 (0.99–1.01)	1.00 (0.99–1.00)	1.00 (0.99–1.00)

*BMI* Body mass index, *CMV* Cytomegalovirus, *UOR* Unadjusted odds ratio, *AOR* Adjusted odds ratio, *CI* Confidence intervals

^a^Weight and height were measured at the study center (see Methods). BMI was calculated using the formula “weight/height^2^” (kg/m^2^)

^b^Blood pressure was measured at the study center (see Methods). Hypertension was defined as a systolic or diastolic blood pressure ≥ 140 mmHg and/or 90 mmHg, respectively

^c^Self-reported information

^d^Results of fractional polynomial regression. Robust independent risk factors (underlined) were defined as those with lower bound 95% CI of AOR not crossing below 1

## Discussion

We examined the prevalence of and risk factors for a weak humoral response to the seasonal adjuvanted influenza vaccine Fluad® in a population-based study among the elderly. Surprisingly, approximately one half and one third of the study participants mounted a weak vaccine response to the influenza A (H1N1) and B strains, respectively.

### Significance of diabetes and herpes zoster as risk factors for a weak vaccine response

A weak humoral response to H1N1 was independently associated with self-reported diabetes, herpes zoster, and (less so) increasing age, i.e. three processes associated with compromised immune function, particularly T cell functionality [24–26]. The fact that the associations were independent of each other indicates that each process by itself affects the vaccine response; however, the final risk likely results from the relative contribution of each the three parameters to immune dysfunction in the particular individual. While increasing age is a known risk factor for an inadequate vaccine response in the elderly [24], findings regarding diabetes are inconsistent. Sheridan et al. found no differences in seroprotection and seroconversion rates of a seasonal non-adjuvanted influenza vaccine between adult patients with and without type 2 diabetes [27]. Furthermore, no differences in the humoral response to a non-adjuvanted influenza vaccine were detected in a sample of clinically well-controlled elderly patients with diabetes mellitus [28]. Rokni et al. hypothesized that failure to detect an association may be explained by good glycemic control, i.e. individuals with well-controlled glycemic status may have a humoral response similar to those of healthy individuals. However, there are no studies comparing humoral vaccine responses between diabetics with good vs. suboptimal glycemic control [29]. In addition, none of the aforementioned studies featured adjuvanted vaccines, and HbA_1C_ (as an objective biomarker of glycemic control) could not be measured in our biobanked study samples as this test is performed on whole blood. Thus, dedicated studies are required to assess the impact of diabetes on vaccine responses to adjuvanted seasonal influenza vaccines in the elderly. Regarding herpes zoster, an association with the humoral response to influenza vaccination has not been reported so far. Of note, in our study it also was a risk factor for a nonresponse to the other influenza A virus component, the H3N2 antigen, further supporting that diabetes and herpes zoster affect the risk of an adequate vaccine response independent of each other. The positive association of diabetes and herpes zoster and an inadequate influenza vaccine response are important findings as the burden of both disorders is highest among elderly individuals. For example, in Germany every fifth individual over 70 years of age has a diagnosis of diabetes [30]. Similarly, the highest incidence of herpes zoster is observed among elderly individuals [31, 32]. Both diabetes mellitus and herpes zoster feature suboptimal T cell function [25, 26] and may also contribute to “inflammaging” [33, 34], a term that describes the chronic progression of a pro-inflammatory status that might hamper the induction of an efficient immune response. Furthermore, an increasing frequency of immunosenescent cells -especially T cells- due to age-related thymic involution may result in reduced vaccine-induced immunity in the elderly [35, 36]. Diabetes and herpes zoster might represent factors that should be considered for the optimal selection/design of specialized vaccination strategies; moreover, both constitute potentially modifiable risk factors. In the case of zoster, this could potentially be achieved by administration of adult VZV vaccination. However, while we cannot rule out a post-zoster immune suppression, it is quite plausible that it is the underlying immune dysfunction and not VZV reactivation per se, that is responsible for the poor vaccine response observed in our study. Further studies investigating additional vaccines against influenza and other infectious diseases are required to address whether diabetes and herpes zoster infection interfere with vaccine non-responsiveness in general.

### Lack of risk factors for a non-response to the influenza B vaccine antigen

We did not find any association between the response to the B strain and the examined variables, and are not aware of any proven immunological reasons for this phenomenon. Based on the aforementioned model that T-cell dysfunction underlies at least part of the non-response to the A antigens, it is tempting to speculate that immune responses against influenza A and B HA antigens differ mechanistically at the level of T-cell responses. Future studies should, therefore, be geared toward a better understanding of differences in immune responses against these antigens. The prevalence of the B non-response in our study was more than twice as high as that of H3N2, for which zoster was identified as a risk factor; therefore, the lack of detection of risk factors for the B non-response was unlikely due to insufficient power. In addition, other variables which were not assessed in our study may play a role, and further studies are clearly needed to search for risk factors for a nonresponse to the B component among elderly individuals in order to optimize protection of this vulnerable age group by future influenza vaccines.

### Lack of impact of BMI and CMV serostatus on vaccine responses to all three antigens

We found no association between the humoral vaccine response and BMI. This is in contrast to other studies which showed an association between increased BMI and/or obesity and a weak immune response to influenza vaccination [37]. However, the findings are inconsistent and other studies showed no association in varied study populations, e.g. HIV-positive individuals [38], health-care workers [39], but also among individuals over 50 years of age [40]. CMV exposure has been postulated to play a considerable role in the development of immunosenescence [41], which in turn may influence the response to vaccination. However, studies of the association between CMV serostatus and the vaccine response are contradictory. Derhovanessian et al. reported that CMV sero-positivity in elderly individuals and the associated accumulation of late-differentiated CD4^+^ T cells correlated with weak influenza vaccine responsiveness [42]. This finding is supported by Trzonkowski and colleagues who found a correlation between CMV sero-positivity and low HAI titers upon influenza vaccination [43]. In contrast, a different study did not find an association of CMV seropositivity and weak influenza vaccine responsiveness in the elderly [44]. Interestingly, McElhaney et al. and Davis et al. found that CMV-positive individuals even mounted a better humoral response to influenza vaccination [45, 46]. The impact of CMV exposure upon influenza vaccine responses may therefore vary considerably depending on factors intrinsic to the study population and/or the vaccine used.

### Strengths and limitations

One of the strengths of this study is the population-based study design with post-stratification weighting aiming to provide nationally representative estimates of the humoral response to an adjuvanted seasonal influenza vaccine among the elderly. Several potential limitations of the study should be considered: a) the overall response rate to the study was low, which may result in selection bias; b) data regarding medical history (e.g. diabetes and herpes zoster) were collected by self-report, which may introduce a recall bias; c) finally, we did not differentiate between type 1 and 2 diabetes mellitus (although according to the epidemiology of these two disorders, most elderly individuals are expected to have type 2). To overcome the first limitation, we oversampled older age groups to achieve the age distribution of the respective age group of the general population and also applied post-stratification weighting. In addition, we have conducted a non-responder survey to examine a potential nonresponse bias and found that the study participants differed from non-participants only slightly in terms of sociodemographic and health-related characteristics [16].

## Conclusions

These results underscore the high prevalence of an inadequate immune response of elderly individuals to seasonal influenza vaccination, even when an adjuvanted vaccine is used. They demonstrate that risk factors for this vaccine failure may vary considerably depending on the influenza vaccine antigen and, furthermore, strongly suggest the need for additional studies to identify determinants of an inadequate vaccine response to the B component, as they remained elusive in our study.

## Additional file

Additional file 1: **Table S1.** Lack of statistically significant differences in complete blood count by vaccine response. (DOCX 16 kb)

## Data Availability

The data used in the study are available from the corresponding author.

## Abbreviations

AOR: Adjusted odds ratio
BMI: Body mass index
BP: Blood pressure
CI: Confidence intervals
CMV: Cytomegalovirus
HAI: Hemagglutination inhibition
RDE: Receptor destroying enzyme
