# Supplementary material for: Self-reported diabetes and herpes zoster are associated with a weak humoral response to the seasonal influenza A H1N1 vaccine antigen among the elderly
Source: BMC Infect Dis. 2019 Jul 23;19:656. doi: 10.1186/s12879-019-4214-x (PMC6651912; doi:10.1186/s12879-019-4214-x)
Supplement: Supplementary file 1 — Table S1. Lack of statistically significant differences in complete blood count by vaccine response. (DOCX 16 kb) [file 12879_2019_4214_MOESM1_ESM.docx]

**Supplementary Table 1.** Lack of statistically significant differences in complete blood count by vaccine response

|  | **H1N1** | | |  | **H3N2** | | |  | **B** | | |
| --- | --- | --- | --- | --- | --- | --- | --- | --- | --- | --- | --- |
|  | Adequate response^a^ | Weak  response^b^ | p value^c^ |  | Adequate response^a^ | Weak  response^b^ | p value^c^ |  | Adequate response^a^ | Weak  response^b^ | p value^c^ |
| WBC count (x 10^9^L) | 6.65±1.71 | 6.68±1.70 | 0.92 |  | 6.62±1.65 | 6.92±2.00 | 0.40 |  | 6.79±1.68 | 6.45±1.73 | 0.18 |
| Neutrophils (x 10^9^L) | 3.93±1.43 | 4.14±1.40 | 0.29 |  | 3.96±1.29 | 4.49±2.00 | 0.07 |  | 4.09±1.38 | 3.94±1.48 | 0.46 |
| Monocytes (x 10^9^L) | 0.59±0.18 | 0.58±0.16 | 0.72 |  | 0.59±0.17 | 0.55±0.14 | 0.22 |  | 0.61±0.17 | 0.55±0.16 | 0.03 |
| Lymphocytes (x 10^9^L) | 1.90±0.56 | 1.88±1.14 | 0.86 |  | 1.91±0.93 | 1.74±0.54 | 0.35 |  | 1.95±1.02 | 1.79±0.61 | 0.24 |
| Erythrocytes (x 10^12^L) | 4.84±0.43 | 4.78±0.38 | 0.28 |  | 4.80±0.41 | 4.84±0.37 | 0.60 |  | 4.80±0.34 | 4.81±0.50 | 0.94 |
| Haemoglobin (g/dL) | 14.63±1.38 | 14.51±1.13 | 0.50 |  | 14.52±1.27 | 14.88±1.19 | 0.16 |  | 14.50±1.10 | 14.69±1.50 | 0.31 |
| Thrombocytes (tsd/µL) | 223.9±54.1 | 227.4±54.3 | 0.65 |  | 226.1±55.5 | 222.2±44.7 | 0.73 |  | 227.3±54.1 | 222.8±54.4 | 0.57 |
| ^a^ An adequate response to the respective components of the influenza vaccine is defined as at least a 4-fold HAI titer increase between day 0 and 21 or as a titer ≤10 on day 0 and ≥40 on day 21 post vaccination.  ^b^ A weak vaccine response was defined as a HAI titer increase <4-fold or <40 for individuals with a titer ≤10 before vaccination.  ^c^ Independent sample t-test | | | | | | | | | | | |
